# Effects of Garlic on Cytochromes P450 2C9- and 3A4-Mediated Drug Metabolism in Human Hepatocytes

**DOI:** 10.3797/scipharm.1002-11

**Published:** 2010-06-09

**Authors:** Beatrice E. Ho, Danny D. Shen, Jeannine S. McCune, Tot Bui, Linda Risler, Ziping Yang, Rodney J. Y. Ho

**Affiliations:** 1 University of Washington Department of Pharmaceutics, 1959 Pacific Street, H-272, Box 357610, Seattle, WA 98195, USA; 2 University of Washington Department of Pharmacy, 1959 Pacific Street, H H-375, Box 357630, Seattle, WA 98195, USA; 3 Fred Hutchinson Cancer Research Center, Clinical Research Division, 1100 Fairview Ave. N. PO Box 19024 Seattle, WA 98109, USA

**Keywords:** Garlic, Cytochrome P450 2C9, Drug Interactions

## Abstract

Several reports suggest garlic supplements may inhibit the metabolism of cytochrome P450 (CYP) 2C9 and CYP3A4 substrates, such as warfarin and saquinavir. To characterize the effects of garlic extract on CYP2C9 and CYP3A4 enzyme activity immortalized human hepatocytes (Fa2N-4 cells) were exposed to garlic extract (0–200 μg/mL). CYP2C9 and CYP3A4 enzyme activities were evaluated in parallel with enzymatic activities, expression of respective RNA transcripts was also assessed.

Exposure to increasing concentrations of garlic extract led to progressive reduction in Fa2N-4 CYP2C9 activity as detected by diclofenac hydroxylation. CYP2C9 mRNA expression also revealed a concentration-dependent reduction. Greater than 90% reduction in CYP2C9 activity was observed following four days of exposure to 50 μg/mL garlic extract. In contrast, exposure to garlic extract had no effect on the CYP3A4 enzymatic activity or RNA transcript concentration in Fa2N-4. Therefore, suppression of CYP2C9 expression and activity is a heretofore unrecognized mechanism by which garlic extract may modulate CYP activity. Exposure of hepatocytes to garlic extract may reduce the expression and activity of CYP2C9 with no detectible effects on CYP3A4.

## Introduction

Garlic supplements are amongst the top ten best selling herbal supplements, which leads to concern over the reported garlic-drug interactions [1–3]. The well-known adverse interaction between garlic and warfarin, a cytochrome P450 (CYP) 2C9 enzyme substrate, is presumably due to the antiplatelet effects of garlic [4]. The administration of enteric coated 2g/dose x twice daily garlic for two weeks did not alter warfarin pharmacokinetics or its effects in healthy male volunteers [5]. Chronic (i.e., greater than 21-days) ingestion of garlic powder leads to increased clearance of saquinavir, a CYP3A4 and p-glycoprotein substrate [6, 7]. These data suggest that garlic may induce both CYP2C9 and CYP3A4 activities, possibly due to activation of shared regulatory mechanisms [e.g. constitutive androstane receptor (CAR)] for their gene transcription.

Two in vitro studies on cell-free enzymes [8, 9] demonstrated that garlic constituents competitively inhibit several recombinant CYP enzymes, namely CYP2C9, CYP2C19 and CYP3A4. Aqueous extracts from odorless garlic products inhibited CYP3A4 (range of inhibition by the six separate products, 30.8–53.8%) to a slightly greater extent than CYP2C9 (range of inhibition: 4.8–33.5%). Steam-distilled garlic oil inhibited CYP2C9 and CYP3A4 to a comparable extent on average, while freeze-dried garlic tablets did not inhibit CYP2C9 activity [8]. Allicin, a major organosulfur constituent of some garlic supplements, was a more potent inhibitor of cDNA-expressed CYP2C9 than CYP3A4 (IC_50_ 5.4 vs. 43.7 μM, respectively) [8]. On the other hand, a human microsomal P450 activity study suggested that only two garlic constituents, *S*-allyl- or *S*-methyl-L-cysteine at 0.1 mg/mL significantly inhibited CYP3A4 with no impact on CYP2C9, CYP2C19 or CYP1A2 [10].

The limitation of cell free microsomes or recombinant enzymes in studying the effect of garlic extract on CYP regulation can be overcome by turning to primary human hepatocytes. Unfortunately, inconsistent availability and the high cost of primary human hepatocytes are major hurdles. Recently, an immortalized human hepatocyte, Fa2N-4 cell line was shown to exhibit characteristics similar to that of primary hepatocytes, particularly induction and inhibition of CYP enzymes. The Fa2N-4 cells are responsive to rifampin’s induction of CYP2C9 and CYP3A4 at a magnitude similar to that of primary human hepatocytes [11]. Therefore, we used the Fa2N-4 cells to evaluate the effects of garlic extract on the expression and activity of CYP2C9 and CYP3A4 in the cells.

## Results and Discussion

Rifampin successfully induced CYP2C9 and CYP3A4 activity and mRNA expression in the Fa2N-4 cells, which confirms the responsiveness of this immortalized cell line to CYP induction (Tables 1 and 2). As shown in Table 1, CYP2C9 activity in Fa2N-4 cells was induced by rifampin to 0.95–2.48 fold compared to media control exposure. Under the same conditions, exposure of Fa2N-4 cells to either 100 or 200 μg/mL garlic significantly reduced CYP2C9 activity in a concentration- and time-dependent manner. By days three and four, both 100 and 200 μg/mL concentrations of garlic lowered CYP2C9 activity to nearly undetectable levels (Table 1). We next replicated the experiment to determine whether at lower garlic concentrations, CYP2C9 activity could be induced (Table 2). We found that at 50 μg/mL, garlic could still suppress hydroxylation of diclofenac by >90% at day four. With respect to CYP3A4 activity, exposure of Fa2N-4 to varying concentrations of garlic extract did not alter CYP3A4 activity (Table 2).

We also determined the effects of garlic on RNA transcripts of CYP2C9 and CYP3A4 in Fa2N-4 cells. The RNA levels were determined on day four using a validated, quantitative RT-PCR assay for each of the target gene transcripts. As shown in Table 2, exposure of these cells to rifampin induces both CYP2C9 and CYP3A4 mRNA transcripts by about four-fold compared to control medium. When the same cells were exposed to garlic, CYP2C9 showed a garlic concentration-dependent reduction in transcript levels. In contrast, exposure to even up to 100 μg/mL of garlic extract did not appear to significantly alter the CYP3A4 transcript concentration in hepatocytes. These results suggest that garlic extract had a selective suppressive effect on the mRNA expression of CYP2C9 in Fa2N-4 hepatocytes. Taken together, the enzymatic activity of CYP2C9 and CYP3A4 in these hepatocytes appeared to have tracked the effects of garlic on RNA transcript levels.

Although it has been suggested that garlic supplements may induce CYP activity in vivo, there is little direct evidence that garlic extract up-regulates human CYP2C9 or CYP3A4 expression. Using a novel immortalized hepatocyte Fa2N-4 cell line that exhibits similar characteristics as those of primary human hepatocytes, we have systematically studied the modulating effects of garlic extract on CYP 2C9 and CYP3A4 mRNA expression and catalytic activity. Our results show that garlic suppresses CYP2C9 expression and activity in a remarkable, concentration-dependent manner, but that garlic has no discernable effect on CYP3A4 within the same cells. The changes in CYP2C9 activity paralleled those of RNA transcript levels. The decrease in transcript concentration is not due to any detrimental effects of garlic extract on cell viability, since expression and activity of CYP3A4 were maintained in the same cells. Also, the degree of induction observed with rifampin in our Fa2N-4 cultures (Table 2) is comparable to that reported by others [11]. In regards to mechanism, the decrease in CYP2C9 transcript could reflect either reduction in transcription or increased RNA degradation, or both. As CYP3A4 transcription is not affected by garlic exposure, it appears that the putative effects of garlic on intracellular transcription or message destabilization are a target-gene specific process. The exact mechanism awaits further investigation. It should be noted that garlic extract could also directly inhibit CYP2C9, but not CYP3A4 activity. Using cDNA expressed recombinant CYP3A4 enzyme, in microsome (cell-free) preparations, Foster et al. have shown that various preparations of garlic extract at a fixed 25 mg/mL concentration inhibited CYP3A4 and CYP2C9 activity [9]. It is not known whether garlic extract at the much lower concentrations employed in our experiments (between 5–200 μg/mL) can directly inhibit CYP2C9 activity (Tables 1 and 2). Nonetheless, it is clear that the activity of CYP3A4 in Fa2N-4 was not affected by the chosen medium concentrations of garlic extract (Table 2).

It has been proposed that garlic supplements’ effects on CYP3A4 in humans is likely to be biphasic in nature; that is, inhibition during the early phase of treatment [8, 9], and simultaneous inhibition and induction following chronic treatment. This hypothesis is supported by the minimal effects of short term exposure (four to five days) to garlic on modifying the area under the concentration-time curve (AUC) of a single dose of ritonavir, a substrate for CYP3A4 [12]. Chronic (i.e., 21-days) administration of dehydrated garlic (Natrol's GarliPure™ caplets BID) significantly reduced the AUC and Cmax of the HIV protease inhibitor saquinavir (a CYP3A4 and P glycoprotein substrate) in nine healthy volunteers [6]. It was proposed that garlic constituents lowered the saquinavir plasma concentrations by inducing the drug-metabolizing enzyme CYP3A4 in the intestine and the liver, and/or the membrane efflux transporter P-glycoprotein at the intestinal mucosa. CYP3A4 activity was not affected by 14-day administration of a different dehydrated garlic product (Kwai garlic supplement, three tablets BID) [13]. Only one study to date has examined the effects of steam-distilled garlic oil on CYP3A4; midazolam hydroxylation, a marker of CYP3A4, was not affected by steam-distilled garlic oil (500 mg TID for 28 days) [14]. It appears that garlic has an equivocal effect on hepatic CYP3A4 that may be substrate-dependent.

Given the remarkable ability for garlic extract to suppress the expression and activity of CYP2C9 in Fa2N-4 cells and previous reports of its direct competitive inhibition of CYP2C9, garlic supplements may interact with drugs that are CYP2C9 substrates with narrow therapeutic indices or notable adverse reactions. For example, celecoxib is extensively metabolized by CYP2C9 [15–17] for which dose-dependent cardiotoxicity has been a concern. In addition to celecoxib, garlic supplements may interact with other drugs metabolized by CYP2C9, such as the antiplatelet drug cilostazol, and the antidiabetic agent glyburide [18].

## Conclusions

We have shown that garlic extract suppresses CYP2C9 activity without affecting CYP3A4 in Fa2N-4 hepatocytes. As the Fa2N-4 cell line is an acceptable model of human primary hepatocytes, these data suggest that the concomitant administration of garlic supplements with CYP2C9 substrates may lower metabolic clearance of the CYP2C9 substrates and potentially precipitate adverse drug reactions.

## Experimental

Rifampin – 3-{[(4-methylpiperazin-1-yl)imino]methyl}rifamycin SV – and diclofenac were purchased from Sigma-Aldrich (St. Louis, MO). 4′-Hydroxydiclofenac was purchased from BD Gentest (Woburn, MA). Midazolam (M-908) and α-hydroxymidazolam (H-902) were purchased from Cerillant (Round Rock, TX). Garlic extract was prepared from a 350 mg Garlicin™ tablet (dehydrated garlic preparation from Nature’s Way, Springville, Utah). The final preparation contained 3,200 μg allicin in 10 mL of phosphate buffered saline at pH 7.4 and was independently verified to be within 5% of the indicated allicin concentration. Three batches of this extract were used to replicate the results. A single batch of stock solution was prepared, aliquots of which were frozen at −80°C pending use. Frozen aliquots were defrosted immediately prior to experiments and discarded after each use. All other reagents were analytical grade or higher. Human liver hepatocyte Fa2N-4 cells and requisite media were purchased from Xenotech (Lenexa, KS).

### Fa2N-4 Cell Studies

According to manufacturer recommendations, six-well plates were seeded with 1.3 × 10^6^ cells/well, and kept in an incubator set at 37°C, 5% carbon dioxide and 95% relative humidity. Plates were exposed to plain media (control), rifampin (positive control) or garlic at indicated concentrations. For multi-day experiments, fresh rifampin- and garlic-containing media were replaced daily. All spike solutions (i.e., garlic, rifampin, diclofenac, midazolam) were prepared in dimethylsulfoxide (DMSO) and added to a final DMSO concentration of 0.1% v/v. Each treatment was replicated four times.

At the indicated time point, media (i.e., garlic, rifampin, or plain media) was removed from cells, the cells were washed with fresh media, and fresh media containing respective concentrations of either rifampin or garlic, and the CYP probe substrates (i.e., diclofenac 100 μM for CYP2C9 and midazolam for CYP3A4) were placed. After six hours of incubation with diclofenac (100 μM), the culture media was removed and an aliquot was analyzed for 4′-hydroxydiclofenac using a modified LC-MS assay procedure based on that described by Mills et al [11]. The formation rate of 4′-hydroxydiclofenac in each well was typically 3–500 pmole/hr. The results for CYP2C9 activity is normalized based on hydroxylation rate of 4′-hydroxy-diclofenac/hr/mg of cell protein, and expressed as the ratio or fold over that observed for control, untreated cells. To assess CYP3A4 activity at the designated day after garlic exposure, the cells were incubated with fresh media containing rifampin or garlic and midazolam (100 μM). After 15 minute incubation, the media was removed and an aliquot was analyzed for α-hydroxymidazolam using a LC-MS analytical method modified from previously published methods [19, 20]. The formation rate of α-hydroxymidazolam in each well was 8–10 pmole/15 min. The results for CYP3A4 activity is normalized based on hydroxylation rate of α-hydroxymidazolam/min/mg of cell protein, and expressed as the ratio or fold over that observed for control, untreated cells. In a separate experiment, the 15 minute incubation time is verified to be within linear range of product formation rate.

### RNA Analysis

For RNA analysis, Fa2N-4 cells were grown on six-well tissue culture plates coated with 4% (w/v) collagen. Total RNA was extracted from cells on day 5 of exposure to plain medium, rifampin or garlic extract using the mini RNeasy kit according to instructions provided by the manufacturer (QIAGEN, Valencia, CA). RNA (100 ng) was subjected to validated real-time polymerase chain reaction (RT-PCR) (ABI, Foster City, CA) assay using ABI Prism 7700 Sequence Detection System [21] to determine the CYP3A4 and CYP2C9 mRNA transcripts, and normalized using human beta-glucuronidase (hGUS) house-keeping control gene transcript. The data were expressed as fold induction compared to RNA collected from control cells treated with plain media. Statistical analysis for increased levels of RNA in samples compared with vehicle-treatment was conducted using the two-sample, unpaired Student's *t* test, with *p* < 0.05 indicating significant differences. Analysis of variance was used for comparisons between multiple treatments (more than two samples); post-hoc paired comparisons were conducted for the purpose of rank ordering multiple inducers.

## Figures and Tables

**Tab. 1.** Effects of Garlic on CYP2C9 activity

**Treatment**	**Diclofenac hydroxylation on day^a^**			

	**1**	**2**	**3**	**4**
Control	1.00 ± 0.07^b^	1.00 ± 0.01	1.00 ± 0.05	1.00 ± 0.04
Rifampin 10 μM	0.95 ± 0.06	1.13 ± 0.04	2.48 ± 0.04	2.14 ± 0.08
Garlic 100 μg/mL	0.54 ± 0.03	0.35 ± 0.08	0.18 ± 0.06	0.04 ± 0.05
Garlic 200 μg/mL	0.26 ± 0.11	0.04 ± 0.28	ND*	ND

a Fa2N-4 cells were exposed to rifampin or garlic at 100 or 200 μg/mL and on indicated days, were exposed to diclofenac for six hours. The CYP2C9 activity was assessed by detecting hydroxylation of diclofenac, and is expressed as fold increase over that of media-treated control;

b Data expressed were the ratio of garlic or rifampin-treated versus control as mean ± one standard deviation of quadruplicate replicates;

* ND = Non detectable level

**Tab. 2.** Effects of Garlic on CYP2C9 and CYP3A4 activity and mRNA expression by day four of treatment^a^

**Treatment^b^**	**CYP2C9**		**CYP3A4**	

	**Activity^b^ (diclofenac hydroxylation)^a^**	**mRNA transcripts^c^**	**Activity^b^ (midazolam hydroxylation)**	**mRNA transcripts^c^**
**Control**	1.00 ± 0.10	1.03 ± 0.24	1.00 ± 0.04	0.07 ± 0.14
**Rifampin 10 μM**	5.74 ± 0.63	4.14 ± 0.54	7.75 ± 0.30	4.11 ± 0.24
**Garlic 5 μg/mL**	0.93 ± 0.20	1.01 ± 0.40	1.06 ± 0.21	1.11 ± 0.41
**Garlic 25 μg/mL**	1.25 ± 0.08	0.93 ± 0.02	1.13 ± 0.10	0.73 ± 0.25
**Garlic 50 μg/ mL**	0.09 ± 0.01	0.33 ± 0.20	1.16 ± 0.10	0.70 ± 0.34
**Garlic 100 μg/mL**	0.17 ± 0.21	0.04 ± 0.02	1.03 ± 0.10	1.12 ± 0.33

a Data expressed were the ratio of garlic or rifampin-treated versus control as mean ± one standard deviation of four replicates;

b Fa2N-4 cells were exposed to rifampin and varying concentrations of garlic for four days. On day four, the cells were exposed to diclofenac for six hours and midazolam for 15 minutes. The fold induction was compared to that of a media-treated control;

c On day four of treatment, cells were removed and real time RT-PCR assay was used to determine the levels of RNA transcript from CYP 2C9 and CYP3A4. The transcript level reflects the enzymatic activity of the hepatocytes;

* ND = Not determined.

## Non-standard abbreviations

(CYP): Cytochrome P450
(CYP2C9): cytochrome P450 2C9
(CYP3A4): cytochrome P450 3A4
(DMSO): dimethylsulfoxide
(LC-MS): liquid chromatography–mass spectrometry
(μM): micromolar
(mg): milligram
(min): minute
(ng/ml): nanogram/milliliter
(RT-PCR): real time polymerase chain reaction
